# Differences in Importance Attached to Drug Effects Between Patients With Type 2 Diabetes From the Netherlands and Turkey: A Preference Study

**DOI:** 10.3389/fphar.2020.617409

**Published:** 2021-02-25

**Authors:** Sonia Roldan Munoz, Douwe Postmus, Sieta T. de Vries, Arna H. Arnardottir, İlknur Dolu, Hans Hillege, Peter G. M. Mol

**Affiliations:** ^1^ Department of Clinical Pharmacy and Pharmacology, University of Groningen, University Medical Center Groningen, Groningen, Netherlands; ^2^ Department of Epidemiology, University of Groningen, University Medical Center Groningen, Groningen, Netherlands; ^3^ Department of Pharmaceutical Safety and Efficacy, Dada Consultancy B.V., Nijmegen, Netherlands; ^4^ Faculty of Health Science, Bartin University, Bartın, Turkey; ^5^ Dutch Medicines Evaluation Board, Utrecht, Netherlands

**Keywords:** type 2 diabetes mellitus, patient preferences, discrete-choice experiment, The Netherlands, Turkey

## Abstract

**Objective:** The aim of this study was to compare the importance that patients with type 2 diabetes mellitus from the Netherlands and Turkey attach to certain drug effects of oral anti-diabetic drugs.

**Methods:** Data were collected through a cross-sectional survey containing demographic questions and a discrete choice experiment assessing preferences for oral anti-diabetic drugs. Adults from the Netherlands and Turkey were included if they had type 2 diabetes mellitus and had received a prescription of an oral anti-diabetic drug in the last 4 months. The oral anti-diabetic drugs in the discrete choice experiment were described in terms of six attributes: effects on HbA1c, cardiovascular diseases, weight change, gastrointestinal adverse drug events hypoglycemic events, and bladder cancer. Multinomial logit models with country as an interaction factor were fitted.

**Results:** In total, 381 patients were included, 199 from the Netherlands and 182 from Turkey. Patients’ preferences toward drug effects varied between the countries. Turkish patients attached the highest importance to reducing the risk of cardiovascular diseases (relative weight: 0.51, 95% CI 0.45–0.55), followed by reducing hypoglycemic events (relative weight: 0.16, 95% CI 0.11–0.22), and reducing gastrointestinal adverse drug events (relative weight: 0.11, 95% CI 0.07–0.18). Patients from the Netherlands attached the highest importance to gastrointestinal ADEs (relative weight: 0.22, 95% CI 0.14–0.39), followed by reducing hypoglycemic events (relative weight: 0.22, 95% CI 0.16–0.25), and reducing the risk of cardiovascular diseases (relative weight: 0.20, 95% CI 0.13–0.23).

**Conclusion:** Patient preferences may differ across countries. Such differences should be acknowledged in regulatory decisions and clinical practice.

## Introduction

The number of people suffering from diabetes mellitus (DM) was estimated to be 415 million worldwide in 2015, and predictions are that more than 600 million people will be affected by 2040 (Zimmet et al., 2016). Prevalence rates vary across countries, but they are increasing globally. A report published by the World Health Organization stated that 6% of the population in the Netherlands and 13% in Turkey had diabetes in 2014 (WHO, 2016).

DM is characterized by having elevated blood glucose (i.e., HbA1c) levels, which leads to an increased risk of developing serious long-term micro- and macrovascular complications (Inzucchi et al., 2012). While a wide range of drugs is available to control glucose, only some have demonstrated cardiovascular (CV) benefit. Moreover, there are other differences in the effects of these drugs for instance with regards to their influence on body weight and adverse drug events (ADEs) (Stein et al., 2013).

Current international guidelines recommend personalizing treatments based on patient characteristics (e.g. age, diabetes duration, comorbidities, or co-medication). However, despite that physicians can personalize treatments based on such characteristics, treatment targets are reached in only about half of the patients (Bohn et al., 2016; Schmieder et al., 2018). This could be due to poor treatment adherence, as adherence to oral anti-diabetic drugs (OADs) is reported to be generally low (Krass et al., 2015).

One way to improve treatment adherence could be basing treatment decisions not only on patient characteristics but also on patient preferences. Since the effects of OADs are not limited to glucose control, patients might also have different preferences toward such additional effects. Considering patient preferences may increase patient’s satisfaction and subsequently, adherence to treatment (Little et al., 2001; Marchesini et al., 2019).

A systematic review of patient preferences studies among people with type 2 DM concluded that weight loss and glucose control were highly important drug effects to patients. However, other included studies also showed that gastrointestinal (GI) ADEs, hypoglycemic events, heart rate or mode of administration were similarly highly important (Purnell et al., 2014). These differences across studies indicate that patient preferences are heterogeneous. The source of such heterogeneity is, however, poorly understood. Since those preference studies were conducted in different countries, country might be an explaining factor of preferences heterogeneity. The aim of this study is to compare the importance that people with type 2 DM from the Netherlands and Turkey attach to certain drug effects of OADs.

## Methods

### Study Design and Data Collection

For this post-hoc study, we used data from a cross-sectional survey study conducted among people with type 2 DM in the Netherlands (Mol et al., 2015) and in Turkey. Patients being 54 years or older in the Netherlands and 18 years or older in Turkey were eligible for inclusion if they had received at least one prescription of an OAD in the last 4 months, and if they gave written consent.

In the Netherlands, patients were identified from pharmacies in the province of Groningen and were contacted by telephone by pharmacy interns to request permission to send them the questionnaire. Patients who granted permission were asked to complete the questionnaire and send it back via pre-paid mail. In Turkey, patients were recruited in the waiting room of general practices in Ankara. Their medical history was checked and those complying with the inclusion criteria were asked to complete the questionnaire during their wait.

The self-administered, paper-based questionnaire contained demographic questions (i.e. age, gender, body mass index (BMI), educational level, experience with ADEs, and diabetes duration) and a discrete choice experiment (DCE) to evaluate the patients’ preferences for drug effects of hypothetical OADs.

In the DCE, the hypothetical OADs were described in terms of six drug effects, or so-called attributes: Influence on HbA1c, influence on the risk of CV diseases, influence on weight change, GI ADEs, number of hypoglycemic events per month, and influence on the risk of bladder cancer. Five of the six attributes varied in three levels, and one attribute varied in two levels (Table 1). A description of how the attributes and levels were selected has been published previously (Mol et al., 2015). Using a D-efficient design, a total of 18 choice sets with two drugs each were created. To reduce the number of questions for each patient, the choice sets were divided into three blocks of six choice sets, and patients were randomized to respond to one of those blocks (Reed Johnson et al., 2013). Participants were asked to imagine being a patient who needed an additional OAD and to choose each time between one of the two presented drugs. Further details on the selection of the attributes, the hypothetical situation, and an example of a choice set have been previously published (Mol et al., 2015).

**TABLE 1:** Attributes and attribute levels.

Attributes	Levels of each attribute
Influence on HbA1c	Decreases from 8.5% to 8.0%^a^
	Decreases from 8.5% to 7.5%
	Decreases from 8.5% to 6.9%
Influence on the risk of CV diseases	An increased risk (4%)^a^
	Unchanged risk (3%)
	A decreased risk (2%)
Influence on weight change	5% weight gain^a^
	No influence on weight
	10% weight loss
GI ADEs	Throughout the use of the drug^a^
	During the first two weeks
	No stomach complaints
Number of hypoglycemic events per month	More than 2 per month^a^
	1 to 2 per month
	None
Influence on the risk of bladder cancer	Increased risk (0.06%)^a^
	Unchanged risk (0.04%)

^a^ Reference level; CV = cardiovascular; GI = gastrointestinal; ADEs = adverse drug events.

### Outcome Variable, Determinant, and Confounders

The outcome variable in this study was the importance that patients attach to the attributes of OADs. The determinant in this study was the country of the patients, i.e. the Netherlands or Turkey. The other patient characteristics obtained via the questionnaire were considered as possible confounders. These were: age (continuous), sex (female/male), BMI (continuous), educational level (secondary school and below/upper-secondary school and above), experience with ADEs (no/yes), and diabetes duration (continuous).

### Data Analyses

Characteristics of the included patients were summarized descriptively and differences between the two countries were tested using Pearson’s χ^2^ tests, t-tests or Mann-Whitney U tests depending on the type of distribution. Only patients who completed all demographic questions and at least one choice set were included in the analyses.

To analyze the overall results of the DCE, a multinomial logit model with dummy-coded levels for each attribute was used. The importance of each attribute level was measured in terms of utility. The reference levels were normalized to have a utility of zero and an increase in the importance of each level was reflected as an increase in utility. Subsequently, the relative importance of each attribute was calculated by the differential between the attribute level with the highest and the lowest utility value (attribute part-worth). These differentials were then normalized to sum to one, with values closer to zero implying lower importance. For these normalized attribute weights, 95% confidence intervals (CI) were obtained through bootstrapping (based on 1,000 resamples with replacement).

To assess whether the preferences varied between Turkish and Dutch respondents, interaction terms between country and the different attribute levels were stepwise included in the multinomial logit model by performing a forward selection procedure with a threshold level of 0.05 for variable inclusion. The relative importance of the attributes together with the 95% CI were calculated per country using the procedures as described above for the overall model.

Finally, the interaction model was adjusted for potential confounding by including the other patient characteristics through forward selection. R version 3.5.1 was used for the statistical analyses, and *p*-values <0.05 were considered statistically significant. Microsoft Excel^®^ 2010 (Microsoft Corp., Redmond, WA, USA) was used for the figures.

## Results

The questionnaire was returned by 409 patients, of whom 28 were excluded (24 due to missing at least one demographic question and four due to missing all choice sets, Supplementary Table 1). Of the included 381 patients, 199 were from the Netherlands (52%), 45% were male, 45% was higher educated, and 19% had ever experienced an ADE. Furthermore, median age was 63 years, average BMI was 29 kg/m^2^, and median diabetes duration was 8 years. Patients from the Netherlands were more often male, were older, were lower educated, had a higher BMI, and more often experienced an ADE than patients from Turkey (Table 2).

**TABLE 2:** Patient characteristics of the included population.

	Total	The Netherlands	Turkey	*p*-value
N (%)	381	199 (52)	182 (48)	
Males, N (%)	173 (45)	106 (53)	67 (37)	<0.001
Median age (IQR)	63 (52–68)	67 (64–71)	52 (47–59)	<0.001
Higher educational level, N (%)	172 (45)	73 (37)	99 (54)	<0.001
Average BMI (SD)	28.7 (4.93)	29.2 (4.83)	28.1 (4.99)	<0.001
Median diabetes duration (IQR)	8 (4–13)	7 (3–13)	9 (5–13)	0.224
Experience of an ADEs, N (%)	72 (19)	46 (23)	26 (14)	<0.001

IQR = interquartile range; BMI = body mass index; SD = standard deviation; ADEs = adverse drug events.

### Preferences of the Overall Study Population

**FIGURE 1:**
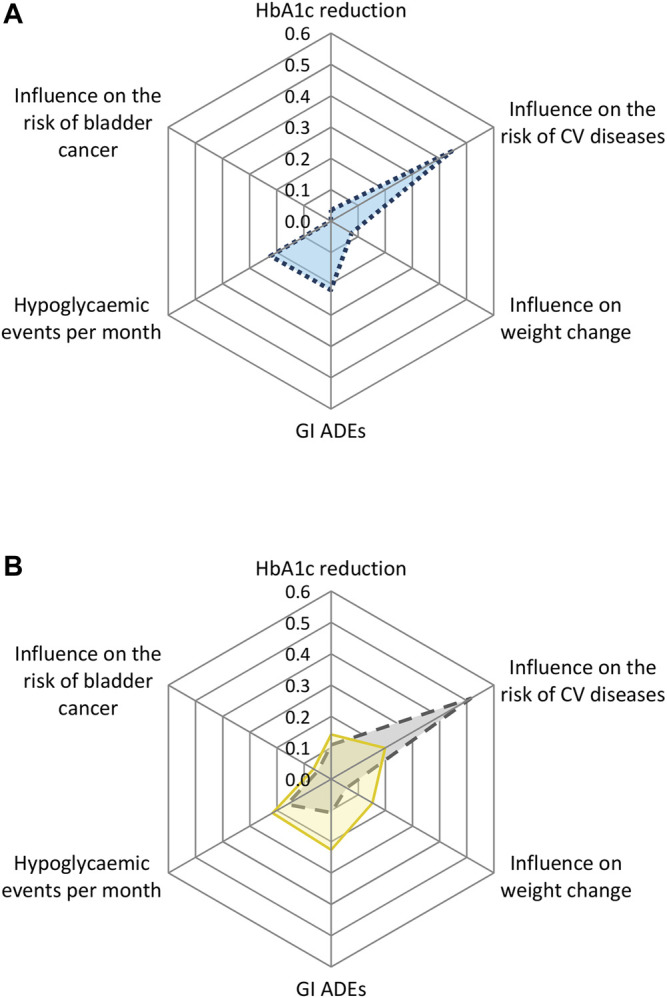
Relative weight scores of the preferences towards the drug attributes of **(A)** the overall study population and **(B)** by country for Dutch and Turkish patients. CV = cardiovascular; GI = gastrointestinal; ADEs = adverse drug events.

The relative weights of the attributes in the overall population are shown in Figure 1A. The largest importance was attached to the attribute influence on the risk of CV diseases. This was followed by the attributes number of hypoglycaemic events per month, GI ADEs, changes in body weight, reduction of HbA1c, and risk of bladder cancer.

**FIGURE 2:**
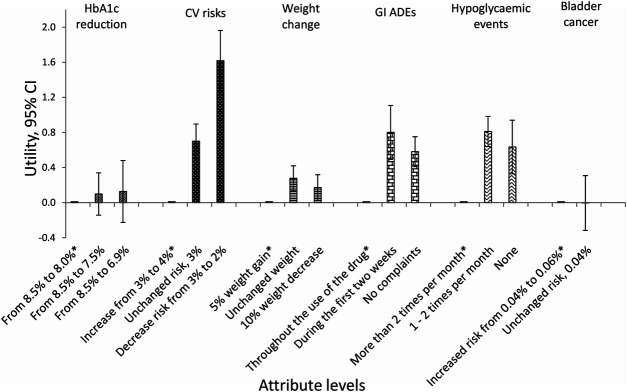
Utility of each attribute level with 95% CI (overall population). *Reference level; CI = confidence interval; CV = cardiovascular; GI = gastrointestinal; ADEs = adverse drug events.

The utility of each attribute level is shown in Figure 2. These results show that the importance of CV diseases is driven by a steep linear increase in utility obtained when reducing the risk of CV diseases from 4% to 2%. For the other attributes, there was less difference in utility between the best and second-best attribute levels (Supplementary Table 2A).

### Association Between Country and Patient Preferences

The analysis with the interaction terms between the attributes and country showed statistically significant differences for the attributes influence on the risk of CV diseases, weight change, GI ADEs, and hypoglycemic events per month (Supplementary Table 2B). Turkish patients attached the highest importance to influence on the risk of CV diseases (relative weight: 0.51, 95% CI 0.45–0.55), followed by hypoglycemic events per month (relative weight: 0.16, 95% CI 0.11–0.22) and GI ADEs (relative weight: 0.11, 95% CI 0.07–0.18). Dutch patients attached the highest importance to GI ADEs (relative weight: 0.22, 95% CI 0.14–0.39) and the number of hypoglycemic events per month (relative weight: 0.22, 95% CI 0.16–0.25), followed by the influence on the risk of CV diseases (relative weight: 0.20, 95% CI 0.13–0.23) and weight change (relative weight: 0.15, 95% CI 0.12–0.20) (Figure 1B, Supplementary Table 3).

After adjusting for the potential confounders, the interaction between attributes and country remained the same. Turkish patients attached more importance to the influence on the risk of CV diseases and Dutch patients to the symptomatic ADEs of GI ADES and hypoglycemic events per month (Supplementary Table 2C).

## Discussion

This study showed that there are substantial differences in drug preferences between patients with type 2 DM from Turkey and the Netherlands. Turkish patients’ choice for an OAD was driven primarily by its effects on reducing the risk of CV diseases whereas Dutch patients’ choice was more multifactorial driven by a desire to prevent symptomatic ADEs (i.e. GI ADEs and hypoglycemic events) in addition to reduce the risk of CV diseases. In both countries, HbA1c reduction and risk of bladder cancer were of limited importance.

There can be various reasons why patient preferences differed across the countries included in our study, such as differences in health behavior and disease risks. For example, Turkish people have been shown to be less physically active, consume more salt and consume more tobacco than people from the Netherlands. (WHO, 2018) In addition, the main cause of death in Turkey is diseases of the circulatory system while in the Netherlands, the main cause of death is cancer (Leening et al., 2014; WHO, 2018). Such differences could make that Turkish patients perceive their risk of CV diseases higher than Dutch people resulting in higher preferences for drugs that reduce this risk in our study.

While Turkish patients’ choices seemed to be mostly driven by the drug’s influence on CV diseases, Dutch patients tended to take into account multiple attributes, mostly symptomatic ADEs in addition to the influence on risks of CV diseases. Differences in preferences across countries has been shown in a previous study where patients with type 2 DM from Germany attached most importance to the risk of GI ADEs and patients from Spain focused mostly on the mode of drug administration (Mansfield et al., 2017). To our knowledge, no previous DCE studies to evaluate preferences about OADs have been conducted among Turkish or Dutch patients with type 2 DM. The results of Dutch patients in our study are similar to a previous study conducted in the Netherlands among patients with type 2 DM, showing, for instance, that hypoglycemia is a major concern for patients with DM and their family members (Nefs and Pouwer, 2018). Our finding that GI ADEs was the most important attribute for Dutch patients is similar to previous studies performed in Germany and the US, which showed higher importance for these ADEs than for other ADEs (Flood et al., 2017; Mansfield et al., 2017). It could be that patient preferences are more similar between countries with a more similar culture and lifestyle. This could also explain why a previous study showed no difference in the most important attribute between patients with type 2 DM in Germany and Sweden (Mohamed et al., 2013). Further research testing similarities and differences across countries would provide explanations for the observed differences.

Patients from both countries considered weight change, HbA1c reduction, and risk of bladder cancer less important. The lack of importance attached to weight change is not in line with the results of a systematic review in which this drug effect was highly important to patients (Purnell et al., 2014). A reason for this difference could be that the mean BMI of the patients in our study was 28.7 kg/m^2^, while several studies included in the systematic review reported mean values of >30 kg/m^2^. The lack of importance regarding HbA1c in our study also differs from previous studies where it was shown to be highly important (Purnell et al., 2014; Hauber et al., 2015; Flood et al., 2017). A plausible explanation for this discordance is that the previous studies used relatively large hypothetical levels of HbA1c reduction, whereas we opted for smaller values achieved with marketed OADs (Mol et al., 2015). The low importance of the risk of bladder cancer in our study could be explained by the low overall risk of this ADE (0.06%), which is in line with a previous study in which severe but rare ADEs were found to be the least important attribute for patients with DM (Donnan et al., 2020).

### Implications

There is a wide range of pharmaceutical and non-pharmaceutical options for the treatment of type 2 DM, and targeting the best treatment for each patient is crucial but also challenging (Cosentino et al., 2020). In general, guidelines for the treatment of type 2 DM state that patient preferences should be taken into consideration. The large differences in preferences between the countries observed in our study highlight the importance of adapting national guidelines to their population as well as international guidelines to reflect differences across countries. OADs are centrally approved in Europe, and the current findings point that even though a drug might not fit preferences of patients from one country, it can have different acceptance in other countries. This might be relevant to discuss at the time of drug approval.

Currently, guideline recommendations to incorporate patient preferences in clinical practice are unspecific, and they are insufficient to guide a healthcare professional on how to detect, discuss or include patient preferences in their treatment decisions (van de Bovenkamp and Zuiderent-Jerak, 2015; Gartner et al., 2019; Cosentino et al., 2020). Although the results of our study are presented at a population level, they can be a step forward in guiding healthcare professionals. A direct implication of our study is that a healthcare professional should be aware of different preferences when treating, for example, a Turkish or Dutch patient. In general, considering patient preferences in treatment decisions can help overcome treatment barriers by making better-informed choices, which would improve adherence and ultimately, treatment outcome (Hauber et al., 2009; Street et al., 2012).

Further studies are needed to assess differences in patient preferences across other countries as well as the role of other patient characteristics in drug preferences.

### Limitations

A general limitation of DCEs is that the results can vary depending on the attributes and levels selected. To address that limitation we used as much as possible values from drugs that are already marketed. A limitation specifically to this study is that Dutch patients were recruited in a more rural area, whereas Turkish patients were recruited in the capital city of Ankara. Including patients from different regions within a country could also have an influence on the results. Future studies assessing differences in preferences across countries should aim to include as similar as achievable populations. Also, 72% of the patients who received the survey in the Dutch population completed it but the response rate for the Turkish population was not available. Furthermore, we adjusted the model for the patient characteristics we had available, but it could be that other relevant characteristics were not taken into account (e.g. medical history).

## Conclusion

This study suggests that patient preferences toward OADs may differ across countries. It was shown that Turkish patients mostly focused on reducing the risk of CV diseases, and Dutch patients mostly focused on reducing short term ADEs as well as reducing the risk of CV diseases. Awareness of heterogeneity in preferences among type 2 DM patients is needed since it can help personalizing treatment, targeting the right treatment for the right patient, improving adherence and ultimately, treatment outcome.

## Data Availability

The dataset used for this study are available upon reasonable request to the corresponding author.

## References

[B1] Bohn B. Schofl C. Zimmer V. Hummel M. Heise N. Siegel E. (2016). Achievement of treatment goals for secondary prevention of myocardial infarction or stroke in 29,325 patients with type 2 diabetes: a German/Austrian DPV-multicenter analysis. Cardiovasc. Diabetol. 15, 72. 10.1186/s12933-016-0391-8 27141979 PMC4855873

[B2] Cosentino F. Grant P. J. Aboyans V. Bailey C. J. Ceriello A. Delgado V. (2020). 2019 ESC Guidelines on diabetes, pre-diabetes, and cardiovascular diseases developed in collaboration with the EASD. Eur. Heart J. 41, 255–323. 10.1093/eurheartj/ehz486 31497854

[B3] Donnan J. R. Johnston K. Chibrikov E. Marra C. A. Aubrey-Bassler K. Najafzadeh M. (2020). Capturing adult patient preferences toward benefits and risks of second-line antihyperglycemic medications used in type 2 diabetes: a discrete choice experiment. Can. J. Diabetes 44, 6–13. 10.1016/j.jcjd.2019.04.014 31311729

[B4] Flood E. M. Bell K. F. de la Cruz M. C. Ginchereau-Sowell F. M. (2017). Patient preferences for diabetes treatment attributes and drug classes. Curr. Med. Res. Opin. 33, 261–268. 10.1080/03007995.2016.1253553 27779433

[B5] Gartner F. R. Portielje J. E. Langendam M. Hairwassers D. Agoritsas T. Gijsen B. (2019). Role of patient preferences in clinical practice guidelines: a multiple methods study using guidelines from oncology as a case. BMJ Open 9, e032483. 10.1136/bmjopen-2019-032483 PMC6924854 31811009

[B6] Hauber A. B. Mohamed A. F. Johnson F. R. Falvey H. (2009). Treatment preferences and medication adherence of people with Type 2 diabetes using oral glucose-lowering agents. Diabet. Med. 26, 416–424. 10.1111/j.1464-5491.2009.02696.x 19388973

[B7] Hauber A. B. Tunceli K. Yang J. C. Gantz I. Brodovicz K. G. Alexander C. M. (2015). A survey of patient preferences for oral antihyperglycemic therapy in patients with type 2 diabetes mellitus. Diabetes Ther. 6, 75–84. 10.1007/s13300-015-0094-2 25586555 PMC4374080

[B9] Inzucchi S. E. Bergenstal R. M. Buse J. B. Diamant M. Ferrannini E. Nauck M. (2012). Management of hyperglycemia in type 2 diabetes: a patient-centered approach: position statement of the American Diabetes Association (ADA) and the European Association for the Study of Diabetes (EASD). Diabetes Care 35, 1364–1379. 10.2337/dc12-0413 22517736 PMC3357214

[B10] Krass I. Schieback P. Dhippayom T. (2015). Adherence to diabetes medication: a systematic review. Diabet. Med. 32, 725–737. 10.1111/dme.12651 25440507

[B11] Leening M. J. Siregar S. Vaartjes I. Bots M. L. Versteegh M. I. van Geuns R. J. (2014). Heart disease in The Netherlands: a quantitative update. Neth. Heart J. 22, 3–10. 10.1007/s12471-013-0504-x 24343132 PMC3890010

[B12] Little P. Everitt H. Williamson I. Warner G. Moore M. Gould C. (2001). Preferences of patients for patient centred approach to consultation in primary care: observational study. BMJ 322, 468–472. 10.1136/bmj.322.7284.468 11222423 PMC26564

[B13] Mansfield C. Sikirica M. V. Pugh A. Poulos C. M. Unmuessig V. Morano R. (2017). Patient preferences for attributes of type 2 diabetes mellitus medications in Germany and Spain: an online discrete-choice experiment survey. Diabetes Ther. 8, 1365–1378. 10.1007/s13300-017-0326-8 29101681 PMC5688991

[B14] Marchesini G. Pasqualetti P. Anichini R. Caputo S. Memoli G. Ponzani P. (2019). Patient preferences for treatment in type 2 diabetes: the Italian discrete-choice experiment analysis. Acta Diabetol. 56, 289–299. 10.1007/s00592-018-1236-6 30306406

[B15] Mohamed A. F. Zhang J. Johnson F. R. Lomon I. D. Malvolti E. Townsend R. (2013). Avoidance of weight gain is important for oral type 2 diabetes treatments in Sweden and Germany: patient preferences. Diabetes Metab. 39, 397–403. 10.1016/j.diabet.2013.06.001 23880594

[B16] Mol P. G. Arnardottir A. H. Straus S. M. de Graeff P. A. Haaijer-Ruskamp F. M. Quik E. H. (2015). Understanding drug preferences, different perspectives. Br. J. Clin. Pharmacol. 79, 978–987. 10.1111/bcp.12566 25469876 PMC4456130

[B17] Nefs G. Pouwer F. (2018). The role of hypoglycemia in the burden of living with diabetes among adults with diabetes and family members: results from the DAWN2 study in The Netherlands. BMC Publ. Health 18, 156. 10.1186/s12889-018-5064-y PMC5774142 29347915

[B18] Purnell T. S. Joy S. Little E. Bridges J. F. Maruthur N. (2014). Patient preferences for noninsulin diabetes medications: a systematic review. Diabetes Care 37, 2055–2062. 10.2337/dc13-2527 24963113 PMC4067391

[B19] Reed Johnson F. Lancsar E. Marshall D. Kilambi V. Mühlbacher A. Regier D. A. (2013). Constructing experimental designs for discrete-choice experiments: report of the ISPOR conjoint analysis experimental design good research practices task force. Value Health 16, 3–13. 10.1016/j.jval.2012.08.2223 23337210

[B20] Schmieder R. E. Tschope D. Tschöpe D. Koch C. Ouarrak T. Gitt A. K. (2018). Individualised treatment targets in patients with type-2 diabetes and hypertension. Cardiovasc. Diabetol. 17, 18. 10.1186/s12933-018-0661-8 29357854 PMC5778654

[B21] Stein S. A. Lamos E. M. Davis S. N. (2013). A review of the efficacy and safety of oral antidiabetic drugs. Expet Opin. Drug Saf. 12, 153–175. 10.1517/14740338.2013.752813 PMC3977601 23241069

[B22] Street R. L. Jr. Elwyn G. Epstein R. M. (2012). Patient preferences and healthcare outcomes: an ecological perspective. Expert Rev. Pharmacoecon. Outcomes Res. 12, 167–180. 10.1586/erp.12.3 22458618

[B23] van de Bovenkamp H. M. Zuiderent-Jerak T. (2015). An empirical study of patient participation in guideline development: exploring the potential for articulating patient knowledge in evidence-based epistemic settings. Health Expect. 18, 942–955. 10.1111/hex.12067 23634973 PMC5060867

[B24] WHO (2016). Diabetes country profiles. Available at: https://www.who.int/diabetes/country-profiles/en/ (Accessed July 24, 2020).

[B25] WHO (2018). Noncommunicable diseases country profiles. Available at: https://www.who.int/nmh/countries/en/ (Accessed July 24, 2020).

[B26] Zimmet P. Alberti K. G. Magliano D. J. Bennett P. H. (2016). Diabetes mellitus statistics on prevalence and mortality: facts and fallacies. Nat. Rev. Endocrinol. 12, 616–622. 10.1038/nrendo.2016.105 27388988

