# Supplementary material for: Differences in Importance Attached to Drug Effects Between Patients With Type 2 Diabetes From the Netherlands and Turkey: A Preference Study
Source: Front Pharmacol. 2021 Feb 25;11:617409. doi: 10.3389/fphar.2020.617409 (PMC7948228; doi:10.3389/fphar.2020.617409)
Supplement: Supplementary file 1 [file table1.pdf]

Supplementary table 1. Missing values for excluded patients

**Patient characteristics**	**The Netherlands (N=226)**	**Turkey (N=183)**	**Total (N= 409)**
Gender	1	0	1
Age	2	0	2
Educational level	8	0	8
BMI	6	0	6
Diabetes duration	11	1	12
**Choice sets**	4	0	4

Some patients missed more than one patient characteristic. Patients excluded in ´Choice sets´ means that they did not respond any of the choice sets. BMI = body mass index
