# Supplementary material for: Differences in Importance Attached to Drug Effects Between Patients With Type 2 Diabetes From the Netherlands and Turkey: A Preference Study
Source: Front Pharmacol. 2021 Feb 25;11:617409. doi: 10.3389/fphar.2020.617409 (PMC7948228; doi:10.3389/fphar.2020.617409)
Supplement: Supplementary file 2 [file table2.pdf]

Supplementary table 2a. Results of the multinomial analyses of the overall population.

Attributes	Attribute levels	Coefficients	Standard error	P-value
HbA1c reduction	HB1*	-	-	-
	HB2	0.10	0.24	0.681
	HB3	0.13	0.35	0.717
Influence on the risk of CV diseases	CV1*	-	-	-
	CV2	0.70	0.19	<0.001
	CV3	1.62	0.35	<0.001
Influence on weight change	WE1*			
	WE2	0.28	0.14	0.054
	WE3	0.17	0.15	0.240
GI ADEs	GI1*	-	-	-
	GI2	0.80	0.31	0.009
	GI3	0.58	0.17	<0.001
Hypoglycaemic events per month	HY1*	-	-	-
	HY2	0.81	0.17	<0.001
	HY3	0.63	0.31	0.038
Influence on the risk of bladder cancer	CA1*	-	-	-
	CA2	0.00	0.31	0.991

HB1= HbA1c decrease from 8.5% to 8.0%*; HB2= HbA1c decrease from 8.5% to 7.5%; HB3= HbA1c decrease from 8.5% to 6.9%; CV1= Increased risk of CV diseases (4%); CV2= Unchanged risk of CV diseases (3%); CV3=Decreased risk of CV diseases (2%); WE1 = 5% weight gain; WE2=No influence on weight; WE3=10% weight loss; GI1 = GI ADEs throughout the use of the drug; GI2= GI ADEs during the first two weeks of treatment; GI3=No GI ADEs; HY1= More than 2 hypoglycaemic events per month; HY2=1 to 2 hypoglycaemic events per month; HY3= No hypoglycaemic events; CA1 = Increased risk of bladder cancer (0,06%); CA2=Unchanged risk of bladder cancer (0.04%). * = Reference level; CV = cardiovascular; GI = gastrointestinal; ADEs = adverse drug events

Supplementary table 2b. Results of the multinomial analyses with interaction by country.

**Attributes**	**Attribute levels**	**Coefficients**	**Standard error**	**P-value**
HbA1c reduction	HB1*	-	-	-
	HB2	0.52	0.28	0.065
	HB3	1.00	0.42	0.018
Influence on the risk of CV diseases	CV2*	-	-	-
	CV2	2.62	0.33	<0.001
	CV3	4.72	0.49	<0.001
Influence on weight change	WE1*	-	-	-
	WE2	-0.01	0.26	0.955
	WE3	-0.52	0.26	0.042
GI ADEs	GI1*	-	-	-
	GI2	0.14	0.45	0.750
	GI3	-0.84	0.28	0.002
Hypoglycaemic events per month	HY1*	-	-	-
	HY2	1.49	0.29	0.000
	HY3	0.84	0.42	0.044
Influence on the risk of bladder cancer	CA1*	-	-	-
	CA2	0.46	0.37	0.221
**Interaction with country**				
	CV2*country	-1.74	0.27	<0.001
	CV3*country	-3.32	0.32	<0.001
	GI2*country	1.13	0.29	<0.001
	GI3*country	2.42	0.25	<0.001
	WE2*country	1.08	0.27	<0.001
	WE3*country	1.37	0.27	<0.001
	HY2*country	-0.74	0.27	0.007
	HY3*country	0.67	0.29	0.021

HB1= HbA1c decrease from 8.5% to 8.0%*; HB2= HbA1c decrease from 8.5% to 7.5%; HB3= HbA1c decrease from 8.5% to 6.9%; CV1= Increased risk of CV diseases (4%); CV2= Unchanged risk of CV diseases (3%); CV3=Decreased risk of CV diseases (2%); WE1 = 5% weight gain; WE2=No influence on weight; WE3=10% weight loss; GI1 = GI ADEs throughout the use of the drug; GI2= GI ADEs during the first two weeks of treatment; GI3=No GI ADEs; HY1= More than 2 hypoglycaemic events per month; HY2=1 to 2 hypoglycaemic events per month; HY3= No hypoglycaemic events; CA1 = Increased risk of bladder cancer (0.06%); CA2=Unchanged risk of bladder cancer (0.04%). * = Reference level; CV = cardiovascular; GI = gastrointestinal; ADEs = adverse drug events

Supplementary table 2c. Results of the multinomial analyses including confounders

**Attributes**	**Attribute levels**	**Coefficients**	**Standard Error**	**P-value**
HbA1c reduction	HB1*	-	-	-
	HB2	0.37	0.29	0.20
	HB3	0.79	0.44	0.074
Influence on the risk of CV diseases	CV2*	-	-	-
	CV2	2.89	0.36	<0.001
	CV3	4.69	0.50	<0.001
Influence on weight change	WE1*	-	-	-
	WE2	0.39	0.30	0.203
	WE3	-0.82	0.29	0.005
GI ADEs	GI1*	-	-	-
	GI2	-0.57	0.49	0.244
	GI3	-1.17	0.30	<0.001
Hypoglycaemic events per month	HY1*	-	-	-
	HY2	1.68	0.33	<0.001
	HY3	0.46	0.46	0.310
Influence on the risk of bladder cancer	CA1*	-	-	-
	CA2	0.72	0.40	0.068
**Interactions with country and confounders**				
	CV2*country	-2.01	0.29	<0.001
	CV3*country	-3.33	0.32	<0.001
	GI2*country	2.24	0.38	<0.001
	GI3*country	3.02	0.29	<0.001
	WE2*country	0.28	0.36	0.441
	WE3*country	1.57	0.36	<0.001

	HY2*country	-1.00	0.37	0.007
	HY3*country	0.90	0.37	0.013
	HB2*diabetes duration	0.02	0.01	0.089
	HB3*diabetes duration	0.04	0.01	<0.001
	CA2*BMI	-0.06	0.02	<0.001
	CA2*age	-0.06	0.01	<0.001
	CV2*GI ADES	-0.35	0.24	0.152
	CV3*GI ADES	-0.56	0.28	0.048
	CA2*educational level	-0.36	0.15	0.019
	CV2*diabetes duration	-0.02	0.01	0.121
	CV3*diabetes duration	0.01	0.01	0.326
	WE2*age	0.04	0.02	0.009
	WE3*age	-0.02	0.02	0.303
	HY2*age	0.04	0.02	0.012
	HY3*age	0.02	0.01	0.244
	HY2*GI ADES	-0.55	0.25	0.030
	HY3*GI ADES	0.02	0.27	0.929
	WE2*GI ADES	0.23	0.26	0.368
	WE3*GI ADES	0.60	0.24	0.013

HB1= HbA1c decrease from 8.5% to 8.0%*; HB2= HbA1c decrease from 8.5% to 7.5%; HB3= HbA1c decrease from 8.5% to 6.9%; CV1= Increased risk of CV diseases (4%); CV2= Unchanged risk of CV diseases (3%); CV3=Decreased risk of CV diseases (2%); WE1 = 5% weight gain; WE2=No influence on weight; WE3=10% weight loss; GI1 = GI ADEs throughout the use of the drug; GI2= GI ADEs during the first two weeks of treatment; GI3=No GI ADEs; HY1= More than 2 hypoglycaemic events per month; HY2=1 to 2 hypoglycaemic events per month; HY3= No hypoglycaemic events; CA1 = Increased risk of bladder cancer (0.06%); CA2=Unchanged risk of bladder cancer (0.04%). * = Reference level; CV = cardiovascular; GI = gastrointestinal; ADEs = adverse drug events; BMI = body mass index
