# Supplementary material for: Differences in Importance Attached to Drug Effects Between Patients With Type 2 Diabetes From the Netherlands and Turkey: A Preference Study
Source: Front Pharmacol. 2021 Feb 25;11:617409. doi: 10.3389/fphar.2020.617409 (PMC7948228; doi:10.3389/fphar.2020.617409)
Supplement: Supplementary file 3 [file table3.pdf]

Supplementary table 3. Relative weights and 95% confidence intervals of the model with interaction by country

**Attributes**	**Country**			
	The Netherlands		Turkey	
	Relative weight	95% CI	Relative weight	95% CI
HbA1c reduction	0.14	0.048 - 0.189	0.11	0.029 - 0.159
Influence on the risk of CV diseases	0.20	0.130 - 0.234	0.51	0.451 - 0.549
Influence on weight change	0.15	0.116 - 0.205	0.06	0.025 - 0.118
GI ADES	0.22	0.139 - 0.386	0.11	0.069 - 0.181
Hypoglycaemic events per month	0.22	0.165 - 0.253	0.16	0.107 - 0.217
Influence on the risk of bladder cancer	0.06	0.004 - 0.135	0.05	0.003 - 0.104

CI = confidence interval; CV = cardiovascular; GI = gastrointestinal; ADEs = adverse drug events
